# Use of FLOSEAL® as a scaffold and its impact on induced neural stem cell phenotype, persistence, and efficacy

**DOI:** 10.1002/btm2.10283

**Published:** 2022-01-21

**Authors:** Hunter N. Bomba, Abigail Carey‐Ewend, Kevin T. Sheets, Alain Valdivia, Morgan Goetz, Ingrid A. Findlay, Alison Mercer‐Smith, Lauren E. Kass, Simon Khagi, Shawn D. Hingtgen

**Affiliations:** ^1^ Division of Pharmacoengineering and Molecular Pharmaceutics UNC Eshelman School of Pharmacy, The University of North Carolina at Chapel Hill Chapel Hill North Carolina USA; ^2^ Department of Neurosurgery The University of North Carolina at Chapel Hill Chapel Hill North Carolina USA; ^3^ Lineberger Comprehensive Cancer Center The University of North Carolina at Chapel Hill Chapel Hill North Carolina USA

**Keywords:** fibrin, gelatin, glioblastoma, neural stem cell, scaffold, TRAIL

## Abstract

Induced neural stem cells (iNSCs) have emerged as a promising therapeutic platform for glioblastoma (GBM). iNSCs have the innate ability to home to tumor foci, making them ideal carriers for antitumor payloads. However, the in vivo persistence of iNSCs limits their therapeutic potential. We hypothesized that by encapsulating iNSCs in the FDA‐approved, hemostatic matrix FLOSEAL®, we could increase their persistence and, as a result, therapeutic durability. Encapsulated iNSCs persisted for 95 days, whereas iNSCs injected into the brain parenchyma persisted only 2 weeks in mice. Two orthotopic GBM tumor models were used to test the efficacy of encapsulated iNSCs. In the GBM8 tumor model, mice that received therapeutic iNSCs encapsulated in FLOSEAL® survived 30 to 60 days longer than mice that received nonencapsulated cells. However, the U87 tumor model showed no significant differences in survival between these two groups, likely due to the more solid and dense nature of the tumor. Interestingly, the interaction of iNSCs with FLOSEAL® appears to downregulate some markers of proliferation, anti‐apoptosis, migration, and therapy which could also play a role in treatment efficacy and durability. Our results demonstrate that while FLOSEAL® significantly improves iNSC persistence, this alone is insufficient to enhance therapeutic durability.

## 1 INTRODUCTION

Glioblastoma (GBM) is an aggressive, stage IV brain cancer and is the most common malignant brain tumor in adults.^1^, ^2^ Current standard of care includes tumor resection, radiation therapy, chemotherapy, with alternating electric field therapy as the most recent clinical advancement for patients.^3^, ^4^ However, complete tumor resection is often unachievable, as GBM is characterized by highly migratory cells that disperse far from the primary tumor mass, often into the contralateral hemisphere.^3^, ^5^ The aggressive, infiltrative nature of GBM results in a high mortality rate and a median patient survival of just 15 months.^1^

To combat the migratory nature of GBM, neural stem cells (NSCs) have been investigated as drug delivery vehicles due to their innate tumor‐tropism. Several preclinical studies have investigated the persistence, migration, and efficacy of immortalized NSCs bearing a range of therapeutic agents in mice.^6^, ^7^, ^8^ Moreover, immortalized, allogeneic NSC therapy has entered human clinical trials for the treatment of GBM (NCT02015819, NCT03072134, NCT01172964, NCT02055196, and NCT02192359).^9^, ^10^, ^11^, ^12^, ^13^ While NSC therapy shows promise, harvesting a sufficient quantity of autologous NSCs is challenging, and immortalized, allogeneic cells pose an immunogenic risk and have potential for unrestrained proliferation.^14^, ^15^ We have improved upon NSC therapy by developing a rapid, single‐transcription factor reprogramming that allows for the direct conversion of fibroblasts to NSCs, known as induced neural stem cells (iNSCs). Here, fibroblasts are isolated from patient skin and stably engineered with lentiviral constructs encoding for the NSC transcription factor *SOX2* and the cytotoxic protein TNFα‐related apoptosis‐inducing ligand (TRAIL). Transduced fibroblasts are then cultured in transdifferentiation media to produce therapeutic, tumor‐homing iNSCs.^16^

While iNSCs are efficacious, persistence in the tumor resection cavity remains a limiting factor. When iNSCs are administered to the resection cavity in saline, more than 50% of iNSCs are cleared by day 10, and nearly all iNSCs are cleared by day 25 postimplantation.^16^ To address this, previous studies have demonstrated that seeding mesenchymal stem cells on both TISSEEL®, a fibrin product, and poly(l‐lactic acid) significantly improve cell persistence compared to cells injected in saline.^17^, ^18^ Additionally, increased persistence of iNSCs has been observed when seeded on Gelfoam®, a gelatin matrix, compared to injection in saline.^19^ However, the optimal characteristics of an iNSC delivery matrix and the relationship between efficacy and increased persistence remain unknown.

Herein, we investigated the use of the FDA‐approved, hemostatic agent, FLOSEAL®, a gelatin and thrombin mixture, as a delivery matrix and its impact on iNSC persistence and efficacy. When subjected to an area with active bleeding, the thrombin component of FLOSEAL® polymerizes with circulating fibrinogen to form fibrin.^20^, ^21^ We postulated that this rapid polymerization would rapidly encapsulate iNSCs, and the gelatin granules would swell to create a physical barrier, thus providing ample protection from the post‐surgical immune response in the resection cavity. We hypothesized that the iNSCs will be able to migrate from the scaffold as FLOSEAL® degrades. It is expected that significant degradation which allows for cell migration will not be observed until the immune response has become less severe. We theorized that the 6‐ to 8‐week resorption timeframe of FLOSEAL® would drastically improve iNSC persistence. In this study, we demonstrate that a FLOSEAL®‐based transplant significantly improves iNSC persistence compared to Gelfoam®, TISSEEL®, and saline injection. The marked increase in persistence lead to improved survival outcomes compared to control‐treated animals using two unique GBM xenograft models, but extensions over therapeutic cells delivered without a scaffold were modest. These results indicate that persistence alone is inadequate as a predictive marker for therapeutic efficacy, and further research is needed to develop the optimal iNSC delivery matrix.

## 2 RESULTS

### 2.1 Encapsulating cells in FLOSEAL®

**FIGURE 1:**
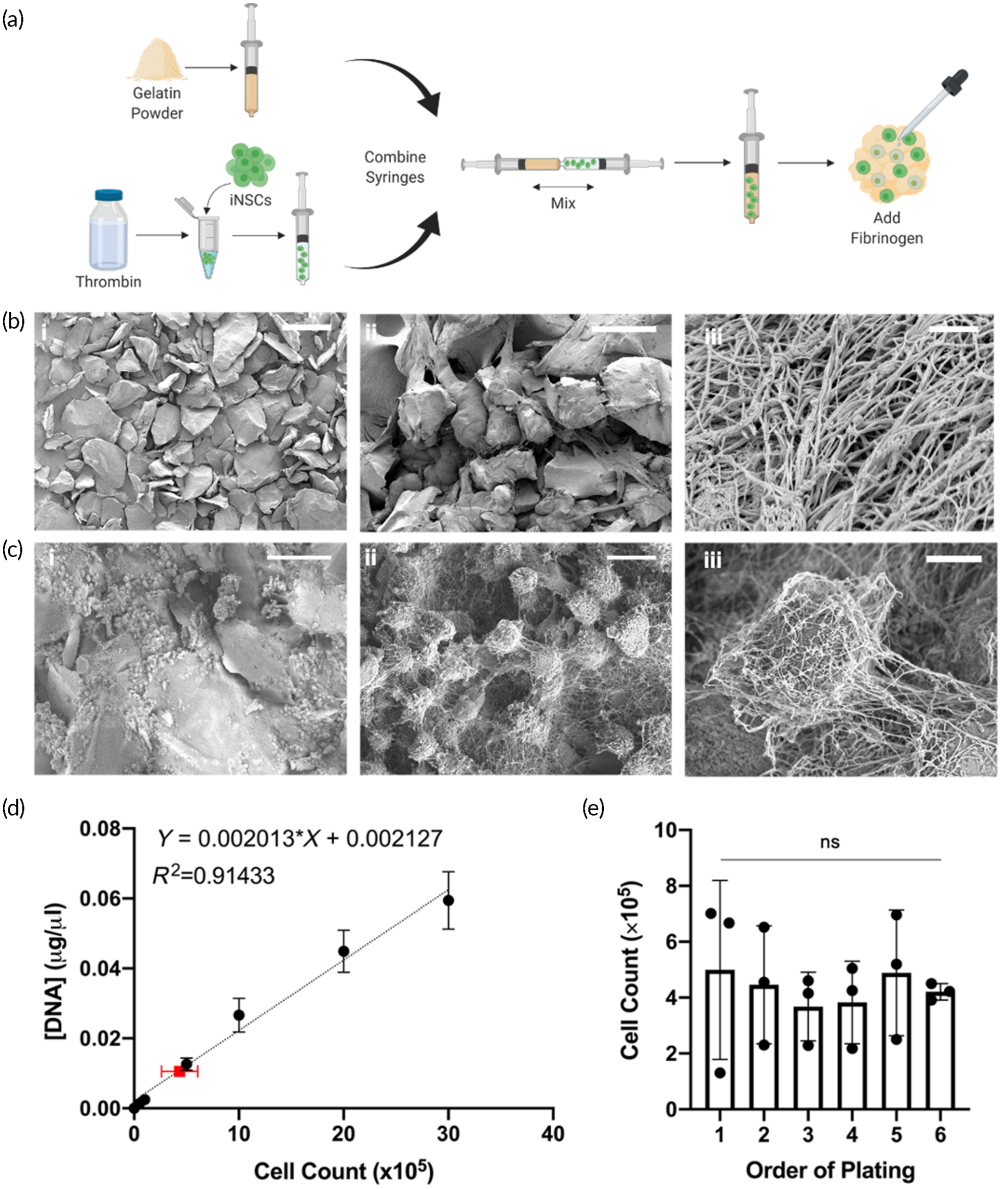
FLOSEAL® material characterization. (a) Schematic depicting FLOSEAL® scaffold fabrication. (b) SEM images of (i) dry gelatin particulate (scale bar, 500 μm), (ii) FLOSEAL® scaffold (scale bar, 200 μm), and (iii) fibrin clot structure (scale bar, 1 μm). (c) SEM images of (i) gelatin, fibrin, and iNSCs (scale bar, 200 μm), (ii) multiple iNSCs entrapped in fibrin (scale bar, 10 μm), (iii) high magnification view of a single iNSC trapped in fibrin (scale bar, 5 μm). (d) Seeding efficiency of fibroblasts in FLOSEAL quantified via DNA concentration. Black points represent standard curve (*n* = 3 per cell density). Red point represents average cell count and DNA concentration of FLOSEAL® samples (*n* = 18). (e) Impact of plating order on the number of cells in each scaffold; all comparisons not significant. Data presented as mean ± standard deviation

Figure 1a depicts the scaffold fabrication process. First, acellular FLOSEAL® scaffolds were observed via SEM. The gelatin particles in the FLOSEAL® kit were found to be heterogeneous in shape and size; on average, particles were determined to be 250 μm at their largest dimension (Figure 1b, i). When combined with thrombin and polymerized with fibrinogen, the gelatin particles become wrapped in a fibrin web (Figure 1b, ii and iii). Similar to the gelatin particles, iNSCs were also found to be encased in the fibrin web, and this cell‐fibrin network encases the gelatin particles (Figure 1c, i–iii).

### 2.2 Seeding efficiency

After understanding the interaction between cells and FLOSEAL®, seeding efficiency, defined as the number of cells calculated to be in the scaffold divided by the theoretical cell count, was determined. FLOSEAL® scaffolds were determined to have a 43.43% ± 17.40% seeding efficiency on average. The total concentration of DNA per sample was 10.6 ng/μl ± 3.5 ng/μl (Figure 1d). Each batch of FLOSEAL® produces six scaffolds contained in a single syringe. To ensure consistency between samples, the seeding variability between scaffolds was also analyzed. The DNA concentration was found to be 12±7, 9±3, 9±2, 10±3, 12±5, and 10±1 ng/μl for scaffolds plated first through sixth, respectively. The total number of cells was calculated to be 4.99 × 10^5^ ± 3.20 × 10^5^, 4.46 × 10^5^ ± 2.11 × 10^5^, 3.68 × 10^5^ ± 1.23 × 10^5^, 3.83 × 10^5^ ± 1.48 × 10^5^, 4.89 × 10^5^ ± 2.25 × 10^5^, and 4.21 × 10^5^ ± 0.30 × 10^5^ for scaffolds plated first through sixth, respectively, using linear regression. Importantly, no statistical significance was observed between any comparison by one‐way ANOVA with Šidák correction (Figure 1e).

### 2.3 Impact of FLOSEAL® on iNSC gene expression

**FIGURE 2:**
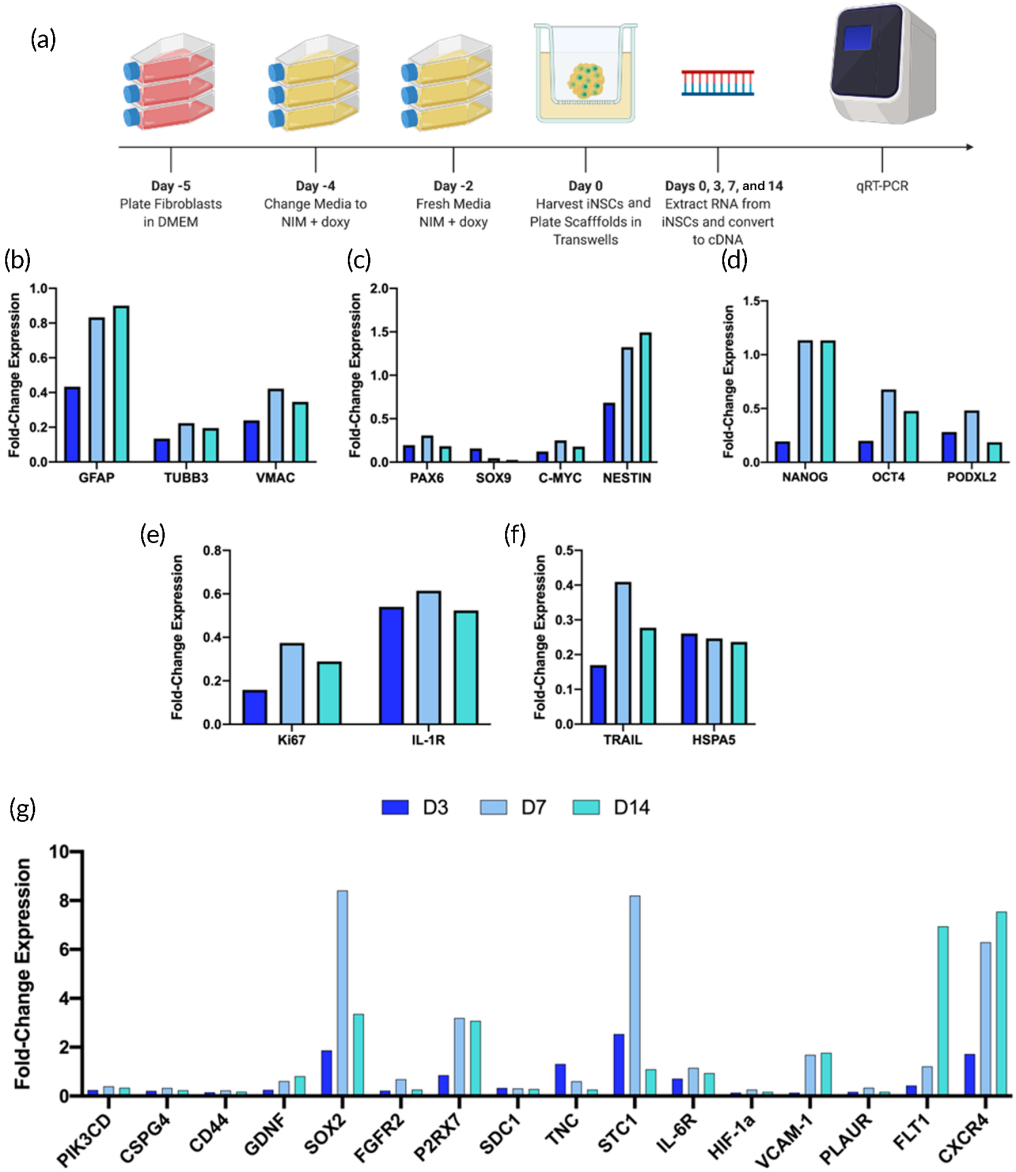
Impact of FLOSEAL® on iNSC phenotype over time. (a) qRT‐PCR experimental design. Gene expression of iNSCs in FLOSEAL® relative to day 0 iNSCs for (b) differentiation, (c) NSC, (d) pluripotency, (e) proliferation, (f) other, and (g) migration markers (five scaffolds pooled per time point)

Previous studies have shown that a material's physiochemical properties can influence gene expression, particularly as it relates to markers of differentiation, proliferation, and migration.^22^, ^23^, ^24^, ^25^ To understand FLOSEAL®'s transcriptomic impact, therapeutic iNSCs were encapsulated in FLOSEAL® and cultured up to two weeks in the matrix using transwell inserts, which allow for scaffold hydration and nutrient exchange but prevents dissolution of the scaffold in liquid media, to study how the material influenced iNSC gene expression (Figure 2a). Cell migration from the scaffold was considered negligible due to the lack of chemoattractant present in the culture system. Gene expression of iNSCs in scaffolds was compared to both non‐transdifferentiated fibroblasts (Supporting Figure S1) and to day 0 iNSCs (Figure 2b–g). “Day 0 iNSCs” denotes fibroblasts that have been transduced and transdifferentiated to become iNSCs but not placed into a scaffold. NSC, differentiation, proliferation, pluripotency, migration, therapy, and anti‐apoptosis markers were monitored; specific genes were selected based on bulk and single‐cell RNA sequencing conducted previously by our group.^26^, ^27^ The differentiation markers, namely *GFAP*, *TUBB3*, and *VMAC*, were found to remain fairly constant in their expression levels over time, but downregulated compared to the day 0 iNSCs (Figure 2b). Of the NSC markers, *NESTIN* was the only one found to be upregulated (Figure 2c). All pluripotency markers were downregulated; however, *NANOG* was virtually unchanged at the day 7 and 14 timepoints (Figure 2d). Interestingly, *Ki67* and *IL‐1R*, two proliferation markers, were also downregulated (Figure 2e). Of note, *TRAIL* and *HSPA5*, an anti‐apoptosis marker, were downregulated (Figure 2f). Lastly, we observed wide variability in the expression of migration markers (Figure 2g). *SOX2*, *P2RX7*, *STC1*, *VCAM‐1*, *FLT‐1*, and *CXCR4* were the most profound upregulations observed.

**FIGURE 3:**
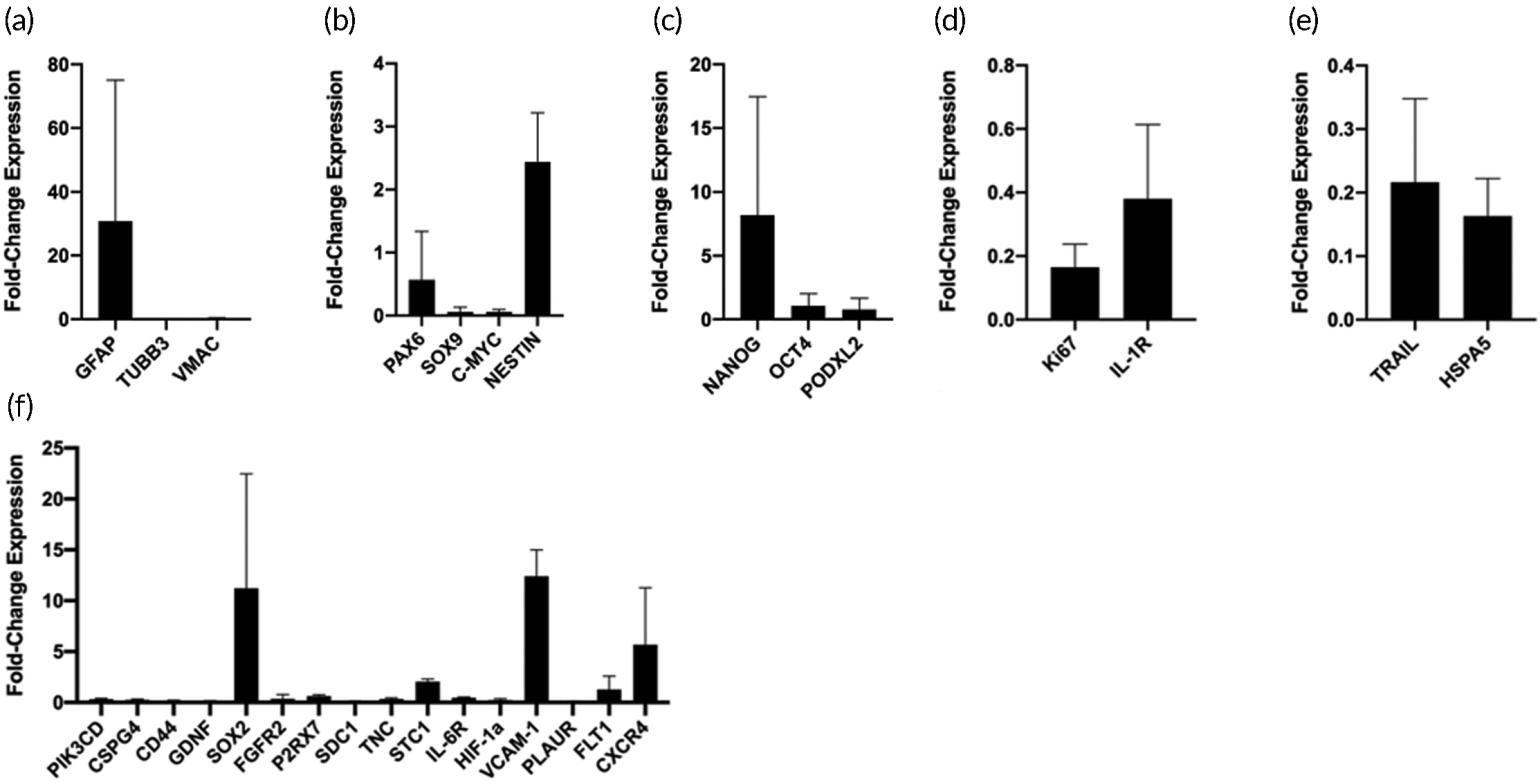
Impact of FLOSEAL® on day 14 iNSC gene expression. Gene expression of iNSCs in FLOSEAL® relative to day 0 iNSCs for (a) differentiation, (b) NSC, (c) pluripotency, (d) proliferation, (e) other, and (f) migration markers (*n* = 3 iNSC batches, six scaffolds pooled per batch)

To further confirm our findings, we opted to repeat this experiment using three unique batches of iNSCs for the day 14 time point. In sharp contrast to the findings presented in Figure 2b, *GFAP* was upregulated while *TUBB3* and *VMAC* remained downregulated (Figure 3a). Similar to the first gene expression experiment, *NESTIN* was the only NSC marker to be upregulated, and similar trends were observed for *NANOG* (Figure 3b,c). The proliferation, anti‐apoptosis, and therapy markers remained downregulated as well (Figure 3d,e). As for the migration genes, similar trends were observed wherein *SOX2*, *STC1*, *VCAM‐1*, *FLT‐1*, and *CXCR4* were upregulated; however, *P2RX7* was slightly downregulated in this experiment (Figure 3f).

### 2.4 In vivo iNSC persistence

**FIGURE 4:**
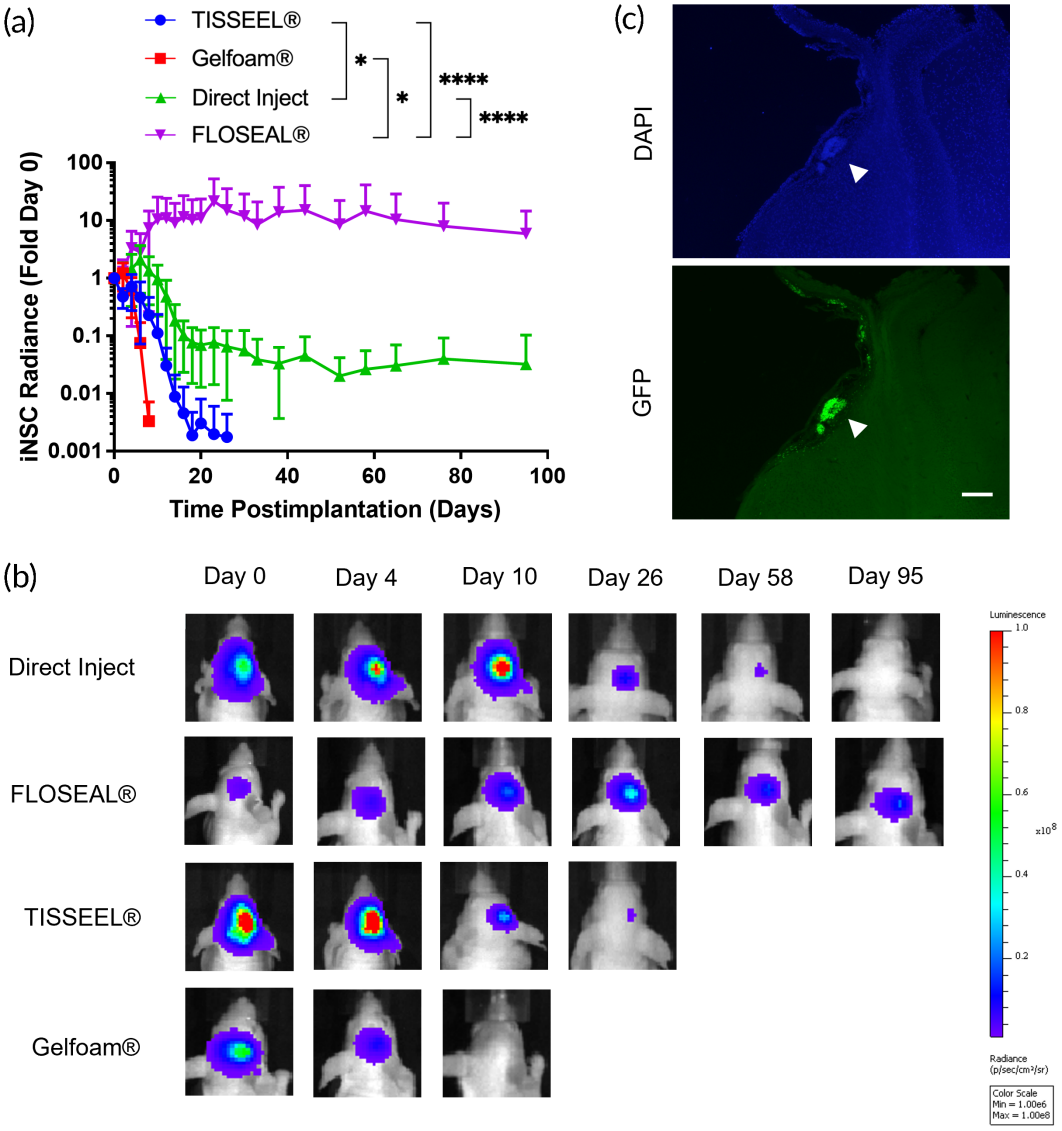
In vivo persistence of cells encapsulated in FLOSEAL®. (a) Fold change in BLI signal of nontherapeutic iNSCs over time (*n* = 5 per group) (* indicates *p* < 0.05; **** indicates *p* < 0.0001). (b) Representative BLI images. (c) Fluorescent images of nontherapeutic iNSCs in FLOSEAL® (arrow) 95 days postimplantation (scale bar, 200 μm)

Next, we sought to compare the persistence of nontherapeutic iNSCs using four different implantation techniques: direct injection or encapsulation in FLOSEAL®, TISSEEL®, or Gelfoam®. Using bioluminescence imaging (BLI), we observed significant differences in Fluc‐tagged iNSC persistence. iNSCs encapsulated in FLOSEAL® persisted significantly longer than iNSCs implanted via direct injection, TISSEEL®, or Gelfoam®. In FLOSEAL®, iNSCs appear to proliferate 21‐fold by day 23 postimplantation and show strong BLI signal 95 days postimplantation. In contrast, iNSCs encapsulated in Gelfoam® and TISSEEL® show near complete clearance on or before day 20 and little proliferation is observed (Figure 4a,b). iNSCs delivered by direct injection in 1X PBS showed near background signal by day 20, also indicating significant clearance. Mice implanted with iNSCs encapsulated in FLOSEAL® were euthanized on day 95, and brains were harvested for analysis. Significantly, iNSCs remained clustered in the mock resection cavity at this time (Figure 4c).

### 2.5 In vivo iNSC efficacy

**FIGURE 5:**
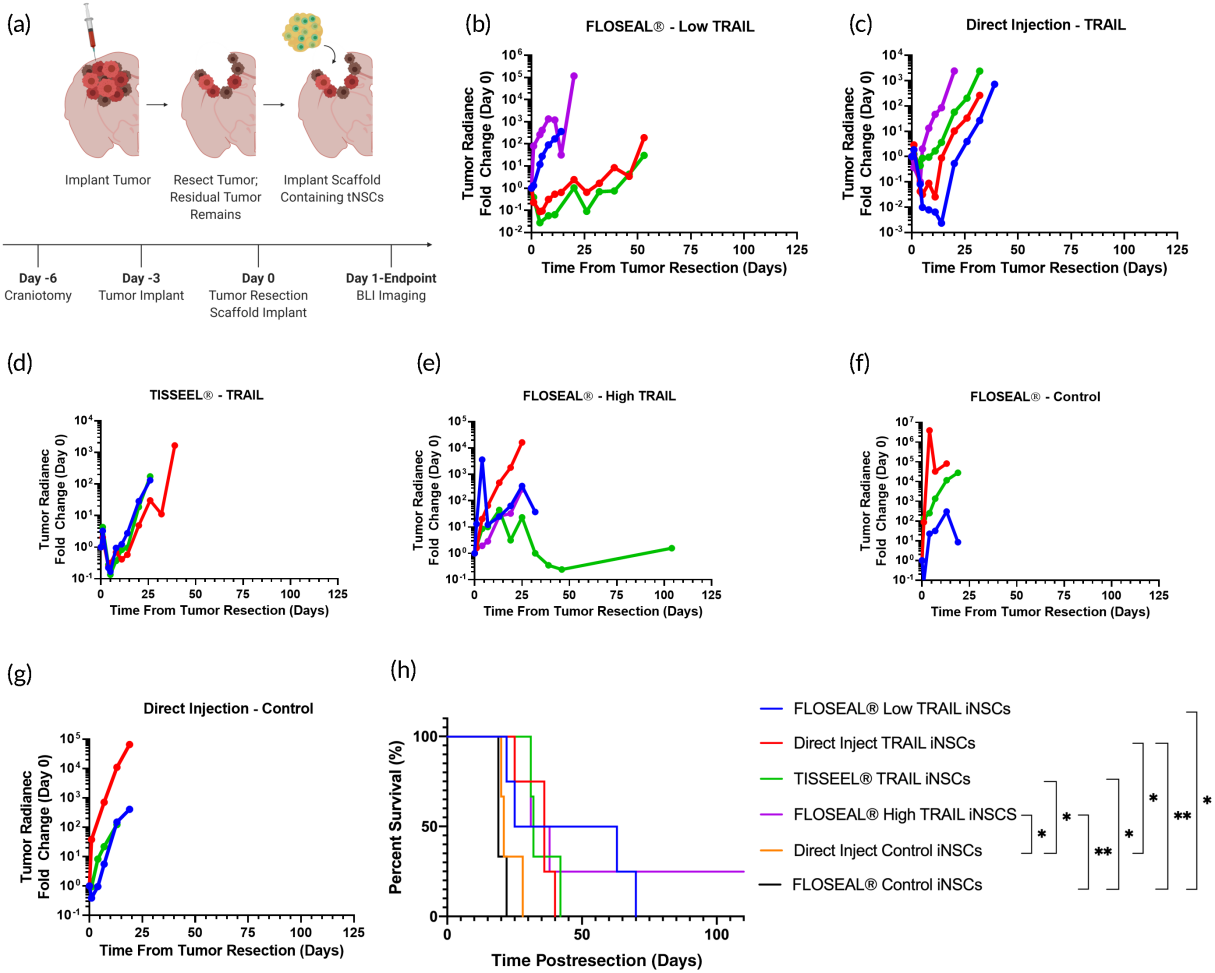
In vivo efficacy of iNSCs encapsulated in FLOSEAL® against GBM8 tumor. (a) Schematic of surgical procedure and timeline. (b) Fold change in GBM8 tumor radiance of mice treated with low dose of TRAIL iNSCs encapsulated in FLOSEAL® (*n* = 4), (c) direct injection of TRAIL iNSCs (*n* = 4), (d) TRAIL iNSCs encapsulated in fibrin (*n* = 3), (e) high dose of TRAIL iNSCs encapsulated in FLOSEAL® (*n* = 4), (f) control, nontherapeutic iNSCs encapsulated in FLOSEAL® (*n* = 3), and (g) direct injection of control, nontherapeutic iNSCs (*n* = 3). Each line represents one mouse. (h) Kaplan–Meier survival curve of mice implanted with GBM8 tumors (* indicates *p* < 0.05; ** indicates *p* < 0.01)

After observing significantly improved iNSC persistence with the aid of FLOSEAL®, we sought to determine if increased persistence correlated to enhanced therapeutic durability and improved survival in mice. We first tested therapeutic durability using the GBM line GBM8. This tumor line was selected for its ability to mimic the invasive nature of tumors seen in the clinic. Three days after implantation, tumors were resected to reflect clinical procedures and treatments were administered into the resection cavity. Tumor size was monitored over time via BLI (Figure 5a). Mice treated with iNSCs encapsulated in FLOSEAL® initially showed steady tumor growth; however, by day 25, some mice began to show decreased tumor growth (Figure 5b,e). The direct injection and TISSEEL® groups showed an initial decrease in tumor volume over the first 5–10 days; however, this was followed by exponential tumor volume increase in the proceeding days (Figure 5c,d). Mice administered nontherapeutic iNSCs displayed rapid tumor growth and significantly shorter lifespans compared to all other groups (Figure 5f,g). In contrast, mice treated with therapeutic iNSCs survived significantly longer compared to mice treated with nontherapeutic iNSCs. The FLOSEAL® low TRAIL (*p* = 0.030), FLOSEAL® high TRAIL (*p* = 0.010), and TISSEEL® TRAIL (*p* = 0.022) groups all survived significantly longer compared to the FLOSEAL® control group. Despite a trend, a significant difference was not observed in survival between the mice treated with a direct injection of therapeutic iNSCs and the FLOSEAL® high TRAIL (*p* = 0.704) or FLOSEAL® low TRAIL groups (*p* = 0.4595).

**FIGURE 6:**
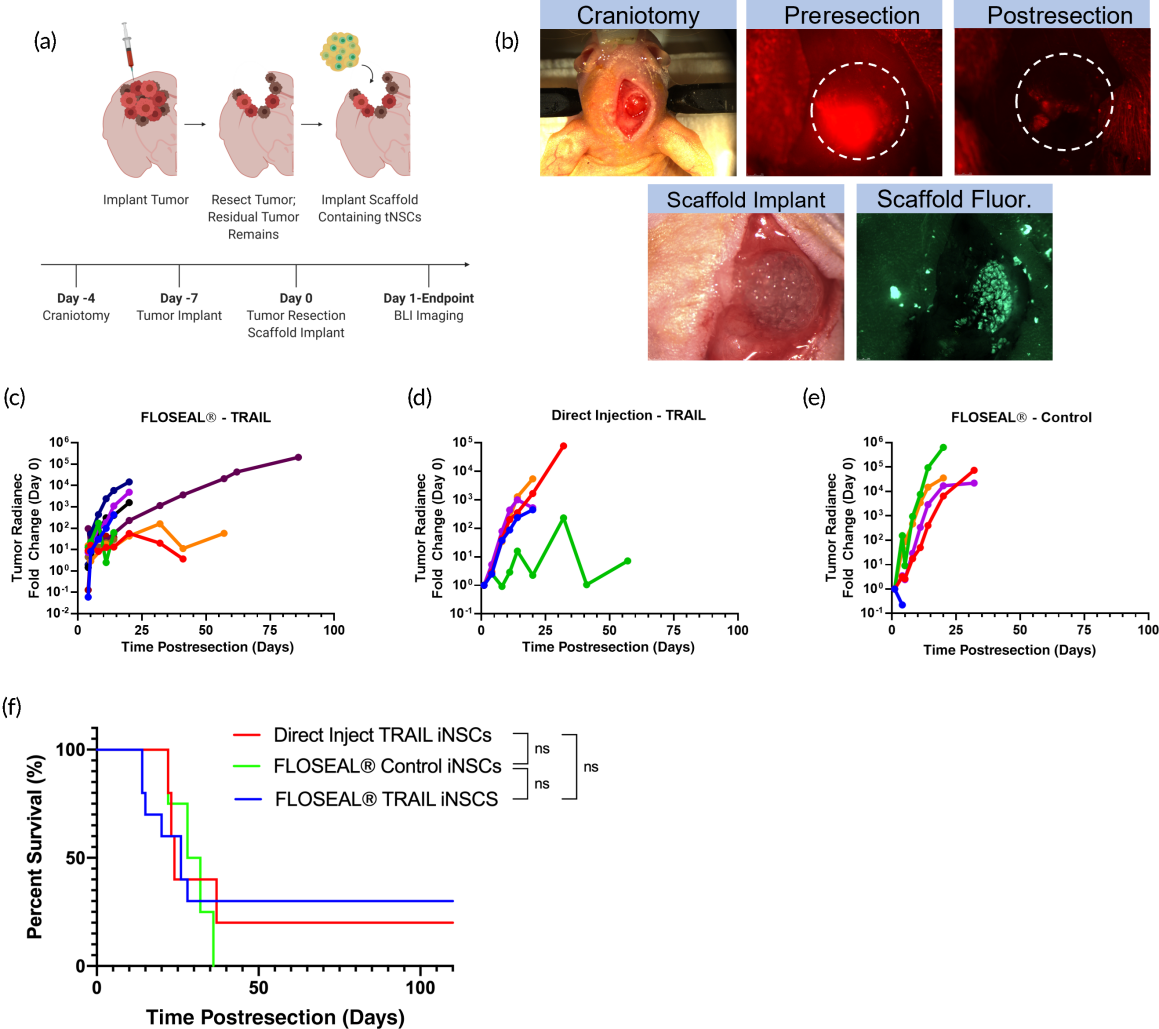
In vivo efficacy of iNSCs encapsulated in FLOSEAL® against U87 tumor. (a) Schematic of surgical procedure and timeline. (b) In vivo surgical images of craniotomy, tumor resection, and FLOSEAL® scaffold implantation. (c) Fold change in U87 tumor radiance of mice treated with TRAIL iNSCs encapsulated in FLOSEAL® (*n* = 10), (d) direct injection of TRAIL iNSCs (*n* = 5), and (e) control, nontherapeutic iNSCs encapsulated in FLOSEAL® (*n* = 5). (f) Kaplan–Meier survival curve of mice implanted with U87 tumors (ns indicates not significant)

We next tested a second GBM tumor model using the U87 cell line to mimic the primary nonmigratory tumor mass seen clinically. Seven days after tumor implantation, the tumors were resected, and iNSC therapies were administered into the resection cavity (Figure 6a). Mice were treated with a direct injection of therapeutic iNSCs in suspension, therapeutic iNSCs encapsulated in FLOSEAL®, or control (nontherapeutic) iNSCs encapsulated in FLOSEAL®. Direct injection control (nontherapeutic) iNSCs were not tested against the U87 model, but exhibited no antitumor effect against the GBM8 model and have not shown any therapeutic effect in our previous works.^27^, ^28^, ^29^ Figure 6b depicts the fluorescence‐guided tumor resection and implant of the scaffold. Mice treated with a direct injection of therapeutic cells exhibited exponential tumor growth following treatment; however, one mouse in this group displayed an initial decrease in tumor volume followed by an increase in tumor volume (Figure 6d). Mice administered therapeutic iNSCs in FLOSEAL® showed different responses within the group. While 70% of mice showed progressive tumor growth within the first 25 days and ultimately succumbed to tumor burden, 30% of mice displayed slower tumor growth and survived beyond 100 days (Figure 6c,f). As anticipated, mice administered nontherapeutic iNSCs encapsulated in FLOSEAL® displayed no tumor suppression (Figure 6e). Despite differences in the tumor progression, no significant differences in survival were observed between any group comparisons.

## 3 DISCUSSION

Survival rates among GBM patients remain extremely poor, which may be attributed to the inadequate standard‐of‐care treatment regimen.^30^ Currently, a major factor in patient survival is extent of surgical resection of the bulk GBM tumor—still, local tumor recurrence almost always occurs.^31^

Novel treatment strategies for GBM are being developed, but often fail before or during clinical trials.^32^ The efficacy of targeted therapeutics remains low due to a lack of known, unique GBM receptors. Immunotherapies are difficult to implement for GBM treatment, as overstimulation is known to cause toxicity, and the blood–brain barrier often prevents the accumulation of recruited immune cells within the tumor.^33^ Additionally, the immune evasive nature of GBM can minimize efficacy. Repetition of treatments is also undesirable—repeated surgery, chemotherapy, and radiation therapy can be particularly harmful to the patient.^32^ The ideal treatment would be administered once yet remain effective until complete tumor eradication is achieved. As such, NSC therapy is a potential alternative to traditional GBM treatment—cells may be administered to the patient at the time of tumor resection, constitutively produce tumoricidal drugs, and remain in the brain while migrating to invasive tumor foci.

Although NSC therapy is a promising new approach for brain cancer treatment, cell persistence remains a challenge to prolonged therapeutic durability, and therefore total elimination of GBM. Our group previously shown the therapeutic promise of iNSC‐mediated delivery of *TRAIL*,^16^, ^18^, ^28^ a cytotoxic peptide that initiates apoptosis through interactions with death receptors 4 (*DR4*) and 5 (*DR5*),^34^ which are highly expressed on GBM tumor cells.^35^ Although *TRAIL* has been investigated as a cancer therapeutic in numerous studies,^36^, ^37^, ^38^ unfavorable pharmacokinetics have prevented its advancement to the clinic. However, constitutive expression of *TRAIL* by iNSCs delivered in the GBM resection cavity can overcome the challenges associated with systemic administration. Cell survival and migration postimplantation are therefore essential to effective tumor‐killing, given the short half‐life of *TRAIL* ^39^ and the highly invasive nature of GBM. To improve the efficacy of the iNSC platform, strategies for enhancing these features must be pursued.

Previous work by our group has demonstrated the benefit of delivery matrices to increase NSC persistence in the tumor resection cavity.^16^, ^17^, ^18^, ^40^ While Gelfoam®, TISEEL®, and HySTEM™ each statistically improved NSC persistence compared to direct inject controls, iNSCs still failed to persist beyond 28 days.^16^, ^17^, ^18^, ^40^ In tumors such as GBM where recurrence is inevitable, there is a strong need to have the iNSCs persist long enough to migrate vast distances across the brain while still constantly producing their therapeutic payload. To achieve this goal, we sought to combine the best features of previous materials, specifically taking into account biocompatibility, cell binding sites, ease of fabrication, and handleability. Based on these criteria, we selected FLOSEAL® as our candidate matrix.

As an FDA‐approved hemostatic product, FLOSEAL®'s intrinsic features make it a desirable scaffold material. The two principal components of FLOSEAL®, gelatin and thrombin, are biocompatible—gelatin as denatured collagen, and thrombin as a critical component of the coagulation cascade.^41^, ^42^ With gelatin and thrombin as the principal ingredients, FLOSEAL® is completely resorbed in patients within 8 weeks and encapsulated NSCs are unlikely to be obstructed by fibrotic encapsulation.^19^, ^43^ For this study, we scaled back the gelatin and thrombin components from the FLOSEAL® kit produce smaller scaffold volumes while keeping the ratio of the components the same as that used clinically. With this formulation, we observed rapid encapsulation of iNSCs in FLOSEAL®. While the gelatin component of FLOSEAL® is rich in RGD sequences, which promotes cell adhesion,^44^ the rapid reaction of thrombin with fibrinogen completely wrapped the iNSCs in a fibrin web with insufficient time to adhere to gelatin particles. This is a possible explanation for the low seeding efficiency of iNSCs in FLOSEAL®, which is also reflected by the lower BLI signals exhibited the FLOSEAL® group in Figure 4.

Furthermore, we observed consistent iNSC density across scaffolds. Because the total volume of gelatin and thrombin (86 mg and 500 μl, respectively) presented here produces enough material to make six scaffolds, we were initially concerned the gelatin particles would be compressed during ejection from the syringe, such that the thrombin and cell solution would be ejected first, therefore producing scaffolds that were initially more liquid and had a higher cell density than those ejected last. Although the first three scaffolds plated were more liquid in nature, no statistical differences were observed in the cell densities, thus ensuring consistent dosing between scaffolds.

Given the volume of literature on the impact of material properties on stem cell proliferation, differentiation, and migration, we sought to understand the impact of FLOSEAL® on iNSC gene expression. In accordance with previous studies, upregulation *NESTIN* and *SOX2* were observed compared to fibroblasts.^16^ Downregulation of *GFAP*, *TUBB3*, and *VMAC* suggest that FLOSEAL® is not inducing differentiation to glial cells or neurons and the iNSCs have transitioned away from a fibroblast phenotype. Surprisingly, *TRAIL* was found to be downregulated at all time points after seeding in FLOSEAL®. However, iNSCs were engineered to secrete *TRAIL* under a constitutive promoter; therefore, additional investigation is needed to elucidate these changes. Interestingly, *KI67* and *IL‐1R* were found to be downregulated. Moreover, downregulation of the anti‐apoptosis gene *HSPA5* was also observed. Despite these findings, we observed a noticeable increase in iNSC signal in our persistence study, indicating that additional signaling factors and interaction in vivo contribute to the iNSC proliferation. Undeniably, there are limitations to this experimental design. The synthetic transwell culture system lacks many components of the in vivo environment that the iNSCs would be exposed to, specifically, cytokines, chemokines, immune cells, and tumor cells.^45^, ^46^ Therefore, due to insufficient external stimuli, it is unsurprising that a number of the migration genes probed were downregulated. However, the substantial upregulation of *SOX2*, *P2RX7*, *STC1*, *VCAM‐1*, *FLT1*, and *CXCR4* suggests that these genes may serve as quality control markers in iNSC therapy optimization and scale‐up. Notably, studies have demonstrated that post‐transcriptional, translational, and degradation processes may play an equally important role in protein expression, and findings elucidated from mRNA should be interpreted with caution.^47^ Future studies will explore changes in iNSC gene expression when encapsulated in FLOSEAL® in immune‐competent mice to understand the impact of the resection cavity microenvironment.

Despite our initial hypothesis that the principal role of gelatin particles would be for iNSC adhesion, gelatin appears to have played a separate role in iNSC persistence. One of the key features of FLOSEAL® as a hemostatic matrix is its ability to expand as a means of compressing and sealing off sources of active bleeding. Based on our remarkable persistence data, we believe the gelatin particles acted as a physical barrier, protecting the iNSCs from immune clearance. While cells persist in Gelfoam® significantly longer compared to previously tested scaffold materials,^48^ we theorize that the lack of persistence of iNSCs in Gelfoam® in the present study is due to poor cell attachment and insufficient penetrance into deeper pores. TISSEEL® also severely underperformed in comparison to FLOSEAL®. Importantly, the BLI signal was confirmed via fluorescence in postmortem brain tissue sections. Given the lack of tumor, and its associated chemokines to promote iNSC migration, it is not surprising to see the iNSCs clustered in the resection cavity after euthanasia on day 95.

Lastly, we investigated if increased iNSC persistence obtained with FLOSEAL® resulted in enhanced therapeutic durability and improved survival outcomes. For these studies, we selected two distinct GBM tumor models, GBM8 and U87. Although both tumors proliferate rapidly in vivo, neither alone fully mimics what is observed clinically; the U87 cell line produces a dense tumor sphere, while the GBM8 cell line results in a diffuse tumor that invades both hemispheres. It is also important to note that GBM8 cells are more sensitive to TRAIL therapy than U87 cells.^49^, ^50^ These attributes explain, at least in part, the differences observed in therapeutic durability and mouse survival observed in the GBM8‐ and U87‐bearing mice. Therapeutic iNSCs injected in suspension into the resection cavity provide immediate tumor suppression, but they lack the persistence to provide a durable response. On the other hand, therapeutic iNSCs encapsulated in FLOSEAL® do not provide immediate tumor suppression, but the FLOSEAL® matrix appears to offer a prolonged therapeutic effect beyond 25 days in select cases. FLOSEAL®'s lack of initial burst release likely explains its inability to significantly reduce tumor volume initially. For all in vivo studies, as noted in the Materials and Methods section, cell densities in FLOSEAL® scaffolds were theoretical calculations made by obtaining a desired cell concentration and assuming equal division among the number of scaffolds made. However, the seeding efficiency data shows that only a 43% seeding efficiency is achieved on average. Therefore, the persistence and efficacy studies were conducted using less than desired cell doses, but despite this, promising trends were observed in therapeutic durability and survival. Based on these results, there is a clear need for a delivery matrix that allows for an initial burst release of iNSCs to combat tumor cells in the immediate vicinity of the resection cavity and sustained release of iNSCs to support therapeutic durability. Future studies will explore how a combinatory burst release and sustained release of iNSCs impacts therapeutic durability as well as the impact of the immune system on iNSC persistence and treatment efficacy.

## 4 CONCLUSIONS

In this study, we show the impact FLOSEAL® has on iNSC gene expression, persistence, and efficacy. While encapsulating iNSCs in FLOSEAL® produced the longest persistence to date, only some mice showed a corresponding increase in survival. Moreover, culturing iNSCs in FLOSEAL® most notably impacted proliferation, anti‐apoptosis, and migration gene expression. These data serve as the framework for future scaffold optimization studies as iNSCs advance toward human clinical trials.

## 5 MATERIALS AND METHODS

### 5.1 Cell lines

U87 tumor cells were obtained from the American Type Culture Collection. U87 cells were cultured in Dulbecco's Modified Eagle Medium (Gibco) containing 10% fetal bovine serum and 1% penicillin/streptomycin (henceforth referred to as standard culture media). GBM8 tumor cells were a gift from H. Wakimoto (Massachusetts General Hospital).^51^ GBM8 cells were cultured in vitro using 500 ml filtered Neurobasal Medium (Gibco) containing 3 mM/L L‐glutamine, 10 ml B27 supplement (Gemini), 2.5 ml N2 supplement (Gemini), 2 μg/ml heparin, 2.5 × 10^4^ U/ml penicillin, 2.5 × 10^4^ μg/ml streptomycin, 62.5 μg/ml amphotericin B, 20 ng/ml fibroblast growth factor, 20 ng/ml epidermal growth factor. Normal human fibroblasts (NHF1s) were obtained from W. Kauffman (University of North Carolina School of Medicine) and were hTERT‐immortalized.

### 5.2 Transduction

Transduction was performed to produce cells expressing optical reporters and therapeutic proteins. Fibroblasts were transduced by incubating the cells with 8 μg/ml polybrene and the lentiviral cocktail for 24 h at 37°C/5% CO_2_. The next day, the virus‐containing media was aspirated and replaced with fresh standard culture media. “Nontherapeutic cells” denotes NHF1 cells transduced with lentiviruses encoding *eGFP‐Fluc*, *SOX2*, and *rtTA*. The *eGFP‐Fluc* plasmid construct contained a puromycin‐resistance gene to allow for selection of cells. “Therapeutic cells” denotes NHF1 cells transduced with *eGFP‐TRAIL*, *Fluc*, *SOX2*, and *rtTA* lentiviruses. GBM8 and U87 cells were transduced using lentiviral *mCh* and *Fluc*. All lentiviruses were purchased from the Duke Viral Vector Core.

### 5.3 iNSC production

To manufacture therapeutic and nontherapeutic iNSCs, 2 × 10^6^ transduced NHF1s were plated in a tissue culture‐treated T‐175 flask using standard culture media. Twenty‐four hours later, the media was changed to STEMdiff Neural Induction Medium (Stem Cell Technologies 05835) supplemented with 2 μg/ml of doxycycline (henceforth referred to as transdifferentiation media). Transdifferentiation media was replaced every other day for 5 days. On the fifth day, cells were harvested using Accutase (STEMCELL Technologies 07922) and passed through a 100 μm filter. Harvested iNSCs were immediately used or kept on ice for no more than 4 h.

### 5.4 Encapsulating iNSCs in scaffolds

To encapsulate iNSCs (Baxter ADS201845), the cells were suspended in 500 μl thrombin from the FLOSEAL® kit and loaded into one of the Luer lock syringes. The second Luer lock syringe was loaded with 86 mg of the gelatin powder. The ratio of thrombin to gelatin powder was determined by taking the total volume and weight of the kit components, respectively, and scaling down to numbers appropriate for murine studies. The two syringes were then connected head‐to‐head and passaged back‐and‐forth twenty times to mix the contents. The contents were left in a single syringe and either used immediately or kept on ice, up to 4 h. This protocol produces approximately 600 μl of cell‐scaffold mixture. For in vitro studies, FLOSEAL® scaffolds were polymerized with 30 μl of fibrinogen from TISSEEL® kits (Baxter 1501653SP). To encapsulate iNSCs in TISSEEL®, 8 μl of fibrinogen was plated into each well of a six‐well plate. Next, iNSCs were suspended in 8 μl thrombin and pipetted directly on to the fibrinogen. TISSEEL® scaffolds were allowed to polymerize for approximately 15 min at room temperature and then kept on ice, up to 4 h. Lastly, to seed cells onto Gelfoam®, a 3 mm diameter hole punch was used to create uniform scaffold discs. Discs were placed into a 96‐well plate and 2.5 μl of the iNSC suspension was pipetted directly onto each side of the disc. iNSCs were allowed to adhere for 1 h at 37°C/5% CO_2_, and were then kept on ice until use, up to 4 h.

### 5.5 Scanning electron microscopy (SEM)

A conjectural count (i.e., not accounting for loss of cells during the seeding process) of 6 × 10^6^ therapeutic iNSCs was encapsulated in FLOSEAL® as described above. The resulting six scaffolds were polymerized and incubated at 37°C/5% CO_2_ for 30 min. Following incubation, scaffolds were submerged in 10% formalin for 30 min. Samples were dehydrated using a graded ethanol series of 50%, 75%, 90%, and 100% ethanol. Next, samples were dried using a critical point drier (Tousimis Autosamdi‐931), placed on aluminum stubs, and sputter coated with 6 nm of gold–palladium (Cressington Sputter Coater 108auto). The seeded scaffolds were imaged using a FEI Helios 600 Nanolab Dual Beam System microscope with a 2 kV accelerating voltage.

### 5.6 In vitro scaffold seeding efficiency

Nontherapeutic NHF1s were harvested using 0.05% trypsin and counted (ThermoFisher Countess II). Next, cells were resuspended to obtain 5 × 10^4^, 1 × 10^5^, 5 × 10^5^, 1 × 10^6^, 2 × 10^6^, and 3 × 10^6^ cells per tube, and genomic DNA was extracted from each tube per manufacturer's protocol (ThermoFisher K182002). DNA was quantified using a Qubit Fluorometric Quantification system (ThermoFisher). Each cell concentration was quantified in triplicate to create a standard curve. To quantify seeding efficiency, nontherapeutic iNSCs were encapsulated in FLOSEAL® as described above, but not polymerized with fibrinogen. The scaffold mixture was divided into six tubes and DNA was isolated. This experiment was done in triplicate to produce a total of 18 scaffold samples.

### 5.7 Quantitative reverse transcription polymerase chain reaction

Therapeutic iNSCs were produced as described above and encapsulated in FLOSEAL® at a conjectural density of 2 × 10^6^ cells/scaffold. Seeded scaffolds were plated on 0.4 μm hanging cell culture inserts (Millipore MCHT06H48) and polymerized as detailed above. To each well, 2 ml transdifferentiation media was added underneath the culture insert, and samples were incubated at 37°C/5% CO_2_; transdifferentiation media was replaced every other day. Five or six samples were pooled on days 3, 7, and 14. Samples were collected by removing the scaffold from the transwell, leaving the insert undisturbed in the case unencapsulated cells were present on the surface. Harvested Therapeutic NHF1s and therapeutic iNSCs not encapsulated in FLOSEAL® (day 0) served as controls. Total RNA was extracted per manufacturer's protocol (ThermoFisher 12183020), immediately converted to cDNA (Invitrogen 11754050), and stored at −80°C until use. Quantitative reverse transcription polymerase chain reaction (qRT‐PCR) was performed using the Applied Biosystems QuantStudio 3 Real‐Time PCR System with TaqMan reagents (ThermoFisher A44360) and custom primer‐probe pairs targeting 30 genes. The thermal protocol used is as follows: UNG incubation at 50°C for 2 min and 1 cycle, enzyme activation at 95°C for 20 s and 1 cycle, denaturing at 95°C for 1 s and 40 cycles, and annealing/extension at 60°C for 20 s and 40 cycles. Relative fold gene expression was calculated using the ΔΔ*C*_*T*_ method using day 0 iNSCs as the biologic control and 18 s rRNA as the endogenous control. Supplemental Table S1 lists genes and corresponding assay IDs.

### 5.8 In vivo iNSC persistence

All animal studies were approved by the Animal Care and Use Committee at the University of North Carolina at Chapel Hill. Six‐ to 8‐week‐old female, athymic nude mice (Animal Studies Core, University of North Carolina‐Chapel Hill) were anesthetized using 2.5% inhaled isoflurane and placed into a stereotaxic frame. The surgical site was prepared using 70% isopropyl alcohol and betadine. An incision was made in the skin on the head of the mouse to expose the skull. Next, using a microdrill, a craniotomy was performed in the right hemisphere, between the bregma and lambda points, on the parietal skull plate. Cold saline and Surgicel® was used to control bleeding. Surgicel® was removed prior to incision closure with Vetbond (3M 1469SB). Postoperative pain was managed with 5 mg/kg of subcutaneous meloxicam 24 h after surgery. Three days following the craniotomy, the mice were anesthetized and prepared for cell implantation. The previous wound was reopened, and the exposed dura mater was removed using an 18 G needle. Using a vacuum pump, a mock resection cavity was created by removing approximately 1 mm^3^ of brain tissue. Next, after bleeding subsided, a conjectural count of 1 × 10^6^ nontherapeutic iNSCs were implanted into the cavity, either in 4 μl of a 1X PBS suspension or encapsulated in a FLOSEAL®, TISSEEL®, or Gelfoam® scaffold. Lastly, the wound was closed with Vetbond, and postoperative pain was managed with 5 mg/kg of subcutaneous meloxicam 24 h after surgery. iNSC persistence was quantified via serial BLI imaging (IVIS Kinetic, Perkin Elmer) using 150 mg/kg D‐luciferin (PerkinElmer 122799) in 1X PBS injected intraperitoneally.

### 5.9 Histology

Mice were anesthetized using 5% inhaled isoflurane. Cardiac perfusion was performed by injecting 5 ml 1X PBS followed by 5 ml 10% formalin into the left ventricle of the heart. Following cervical dislocation, brains were harvested and immediately fixed by transferring into a vial containing 10% formalin. Samples were fixed overnight at 4°C and then transferred to a vial containing 30% sucrose in 1X PBS. Samples were kept at 4°C until the tissue sunk. Next, brains were prepared for cryosectioning by placing the tissue in a cryomold (Tissue‐Tek Cryomold, Sakura), covering with optimal cutting temperature compound (OCT), and freezing at −80°C. Tissue sections were cut 40 μm thick onto microscope slides. Then, OCT was washed away by incubating the sample in 1X PBS at room temperature for 5 min. To visualize cells, the sample was stained with DAPI (Invitrogen) and mounted on ProLong™ Gold Antifade Mountant (Invitrogen).

### 5.10 In vivo iNSC efficacy

Mice were anesthetized using 2.5% inhaled isoflurane and placed into a stereotaxic frame. The surgical site was prepared using 70% isopropyl alcohol and betadine. An incision was made in the skin on the head of the mouse to expose the skull. Next, using a microdrill, a craniotomy was performed in the right hemisphere, between the bregma and lambda points, on the parietal skull plate. Cold saline and Surgicel® were used to control bleeding. Surgicel® was removed, and the wound was closed with Vetbond (3M 1469SB). Three days after the craniotomy, mice were again anesthetized and prepared for surgery. The wound was reopened, and using a stereotaxic auto‐injector, 1 × 10^5^ U87‐mCh‐Fluc cells or 3 × 10^5^ GBM8‐mCh‐Fluc cells suspended in 3 μl of 1X PBS were infused into the brain parenchyma at stereotaxic coordinates 2.5, −0.5, −0.5 from bregma at a rate of 1 μl/min, avoiding the lateral ventricles. Cells were given 5 min to settle before slowly removing the syringe. The wound was closed with Vetbond. Seven days after implanting the U87 tumors and 3 days after implanting the GBM8 tumors, mice were anesthetized and prepared for surgery. The wound was reopened, and the tumors were resected using fluorescence guidance and a vacuum pump. Once bleeding subsided, therapeutic iNSCs were implanted into the cavity in a 1X PBS suspension or encapsulated in FLOSEAL®. For the GBM8 efficacy study where two doses of iNSCs were tested, “FLOSEAL® high” TRAIL denotes mice that received 1.5 × 10^6^ therapeutic iNSCs, and “FLOSEAL® low TRAIL” denotes mice that received 6 × 10^5^ therapeutic iNSCs conjecturally. All other groups received 1 × 10^6^ therapeutic or nontherapeutic iNSCs, again noting conjecturally for the “FLOSEAL® Control iNSC” group. In the U87 efficacy study, FLOSEAL® TRAIL denotes mice that received 1.5 × 10^6^ therapeutic iNSCs conjecturally, and all remaining groups received 1 × 10^6^ therapeutic or nontherapeutic iNSCs again noting conjecturally for the “FLOSEAL® Control iNSC” group. Postoperative pain was managed with 5 mg/kg of subcutaneous meloxicam 24 h after surgery. iNSC Tumor volume was monitored over time via BLI (AMI HTX, Spectral Instruments Imaging). Animals were euthanized when more than 20% of their original body weight was lost or when the animal displayed physical symptoms of pain‐based dehydration, hunched position, tremors, and cold body temperature.

### 5.11 Statistical analysis

Replicate number is defined by n in figure legends. All data presented as mean ± standard deviation unless otherwise stated. Seeding efficiency data analyzed via one‐way ANOVA with Šidák's multiple comparisons test. iNSC persistence data analyzed via one‐way ANOVA mixed effects analysis with Šidák's multiple comparisons test. Survival curves analyzed via log‐rank (Mantel–Cox) test. In all graphs, * indicates *p* < 0.05, ** indicates *p* < 0.01, *** indicates *p* < 0.001, and **** indicates *p* < 0.0001. Statistical analyses were conducted using Prism GraphPad (version 7).

## AUTHOR CONTRIBUTIONS

**Hunter Nicole Bomba:** Conceptualization (lead); data curation (lead); investigation (lead); methodology (lead); writing – original draft (lead); writing – review and editing (supporting). **Abigail Carey‐Ewend:** Investigation (supporting); writing – original draft (supporting). **Kevin T. Sheets:** Conceptualization (lead); investigation (supporting); methodology (supporting); writing – original draft (supporting). **Alain Valdivia:** Investigation (supporting). **Lauren Kass:** Writing – review and editing (lead). **Morgan Goetz:** Investigation (supporting). **Ingrid A. Findlay:** Investigation (supporting). **Alison Mercer‐Smith:** Methodology (supporting); writing – original draft (supporting). **Simon Khagi:** Conceptualization (supporting); resources (supporting). **Shawn D. Hingtgen:** Conceptualization (lead); methodology (supporting); writing – original draft (supporting).

## CONFLICT OF INTEREST

Shawn D. Hingtgen has an ownership interest in Falcon Therapeutics, Inc., which has licensed aspects of iNSC technology from the University of North Carolina at Chapel Hill. Hunter N. Bomba, Abigail Carey‐Ewend, Kevin T. Sheets, Alain Valdivia, Morgan Goetz, Ingrid A. Findlay, Lauren E. Kass, and Simon Khagi have no competing interests to disclose.

### PEER REVIEW

The peer review history for this article is available at https://publons.com/publon/10.1002/btm2.10283.

## Supporting information


**Appendix** **S1**: Supporting information

## Data Availability

All data needed to evaluate the conclusions made in this paper are presented in the paper and the Supplemental Materials. Additional data may be requested from the authors.

## References

[1] Pisapia DJ . The updated World Health Organization glioma classification cellular and molecular origins of adult infiltrating gliomas. Arch Pathol Lab Med. 2017;141:1633‐1645. doi:10.5858/arpa.2016-0493-RA 29189064

[2] Davis ME . Glioblastoma: overview of disease and treatment. Clin J Oncol Nurs. 2016;20(5 Suppl):S2‐S8. doi:10.1188/16.CJON.S1.2-8 PMC5123811 27668386

[3] Alifieris C , Trafalis DT . Glioblastoma multiforme: pathogenesis and treatment. Pharmacol Ther. 2015;152:63‐82. doi:10.1016/j.pharmthera.2015.05.005 25944528

[4] Mittal S , Klinger NV , Michelhaugh SK , Barger GR , Pannullo SC , Juhász C . Alternating electric tumor treating fields for treatment of glioblastoma: rationale, preclinical, and clinical studies. J Neurosurg. 2018;128(2):414‐421. doi:10.3171/2016.9.JNS16452 28298023 PMC6836465

[5] Holland EC . Glioblastoma multiforme: the terminator. PNAS. 2000;97(12):6242‐6244. 10841526 10.1073/pnas.97.12.6242 PMC33993

[6] Aboody KS , Brown A , Rainov NG , et al. Neural stem cells display extensive tropism for pathology in adult brain: evidence from intracranial gliomas. PNAS. 2000;97(23):12846‐12851. doi:10.1073/pnas.97.23.12846 11070094 PMC18852

[7] Aboody KS , Najbauer J , Metz MZ , et al. Neural stem cell‐mediated enzyme/prodrug therapy for glioma: preclinical studies. Sci Transl Med. 2013;5(184):184ra59. doi:10.1126/scitranslmed.3005365 PMC3864887 23658244

[8] Kim SK , Kim SU , Park IH , et al. Human neural stem cells target experimental intracranial medulloblastoma and deliver a therapeutic gene leading to tumor regression. Clin Cancer Res. 2006;12(18):5550‐5556. doi:10.1158/1078-0432.CCR-05-2508 17000692

[9] ClinicalTrials.gov . National Library of Medicine Identifier NCT02015819 (US). Genetically modified neural stem cells, flucytosine, and leucovorin for treating patients with recurrent high‐grade gliomas.

[10] ClinicalTrials.gov . National Library of Medicine Identifier NCT02192359 (US). Carboxylesterase‐expressing allogeneic neural stem cells and irinotecan hydrochloride in treating patients with recurrent high‐grade gliomas.

[11] ClinicalTrials.gov . National Library of Medicine Identifier NCT02055196 (US). Genetically modified stem cells and irinotecan hydrochloride in treating patients with recurrent high‐grade gliomas.

[12] ClinicalTrials.gov . National Library of Medicine Identifier NCT01172964 (US). A pilot feasibility study of oral 5‐fluorocytosine and genetically‐modified neural stem cells expressing *E. coli* cytosine deaminase for treatment of recurrent high grade gliomas.

[13] ClinicalTrials.gov . National Library of Medicine Identifier NCT03072134 (US). Neural stem cell based virotherapy of newly diagnosed malignant glioma.

[14] Aleynik A , Gernavage KM , Mourad YS , et al. Stem cell delivery of therapies for brain disorders. Clin Transl Med. 2014;3(1):24. doi:10.1186/2001-1326-3-24 25097727 PMC4106911

[15] Bovenberg MSS , Degeling MH , Tannous BA . Advances in stem cell therapy against gliomas. Trends Mol Med. 2013;19(5):281‐291. doi:10.1016/j.molmed.2013.03.001 23537753

[16] Bagó JR , Okolie O , Dumitru R , et al. Tumor‐homing cytotoxic human induced neural stem cells for cancer therapy. Sci Transl Med. 2017;9(375):eaah6510. doi:10.1126/scitranslmed.aah6510 28148846 PMC6719790

[17] Bagó JR , Pegna GJ , Okolie O , Mohiti‐Asli M , Loboa EG , Hingtgen SD . Fibrin matrices enhance the transplant and efficacy of cytotoxic stem cell therapy for post‐surgical cancer. Biomaterials. 2016;84:42‐53. doi:10.1016/j.biomaterials.2016.01.007 26803410 PMC4790749

[18] Bagó JR , Pegna GJ , Okolie O , Mohiti‐Asli M , Loboa EG , Hingtgen SD . Electrospun nanofibrous scaffolds increase the efficacy of stem cell‐mediated therapy of surgically resected glioblastoma. Biomaterials. 2016;90:116‐125. doi:10.1016/j.biomaterials.2016.03.008 27016620 PMC5376347

[19] European Medicines Agency . Medical device: floseal hemostatic matrix (VH S/D). Consultation procedure public assessment report (CPAR).

[20] FLOSEAL Hemostatic matrix, 5 mL instructions for use.

[21] Davie EW , Fujikawa K , Kisiel W . Perspectives in biochemistry the coagulation cascade: initiation, maintenance, and regulation. Biochemistry. 1991;30(43):10363‐10370. 1931959 10.1021/bi00107a001

[22] Menon NV , Chuah YJ , Phey S , et al. Microfluidic assay to study the combinatorial impact of substrate properties on mesenchymal stem cell migration. ACS Appl Mater Interfaces. 2015;7(31):17095‐17103. doi:10.1021/acsami.5b03753 26186177

[23] Pathak A , Kumar S . Independent regulation of tumor cell migration by matrix stiffness and confinement. Proc Natl Acad Sci USA. 2012;109(26):10334‐10339. doi:10.1073/pnas.1118073109 22689955 PMC3387066

[24] Charrier EE , Pogoda K , Wells RG , Janmey PA . Control of cell morphology and differentiation by substrates with independently tunable elasticity and viscous dissipation. Nat Commun. 2018;9(1):1‐13. doi:10.1038/s41467-018-02906-9 29386514 PMC5792430

[25] Saha K , Keung AJ , Irwin EF , et al. Substrate modulus directs neural Stem cell behavior. Biophys J. 2008;95(9):4426‐4438. doi:10.1529/biophysj.108.132217 18658232 PMC2567955

[26] Jiang W , Yang Y , Mercer‐Smith AR , et al. Development of next‐generation tumor‐homing induced neural stem cells to enhance treatment of metastatic cancers. Sci Adv. 2021;7(24):eabf1526. doi:10.1126/sciadv.abf1526 34108203 PMC8189583

[27] Buckley A , Hagler SB , Lettry V , et al. Generation and profiling of tumor‐homing induced neural stem cells from the skin of cancer patients. Mol Ther. 2020;28(7):1614‐1627. doi:10.1016/j.ymthe.2020.04.022 32402245 PMC7335733

[28] Bagó JR , Alfonso‐Pecchio A , Okolie O , et al. Therapeutically engineered induced neural stem cells are tumour‐homing and inhibit progression of glioblastoma. Nat Commun. 2016;7(1):10593. doi:10.1038/ncomms10593 26830441 PMC4740908

[29] Okolie O , Irvin DM , Bago JR , et al. Intra‐cavity stem cell therapy inhibits tumor progression in a novel murine model of medulloblastoma surgical resection. PLoS One. 2018;13(7):e0198596. doi:10.1371/journal.pone.0198596 29990322 PMC6038981

[30] Wick W , Osswald M , Wick A , Winkler F . Treatment of glioblastoma in adults. Ther Adv Neurol Disord. 2018;11:1756286418790452. doi:10.1177/1756286418790452 30083233 PMC6071154

[31] Rapp M , Baernreuther J , Turowski B , Steiger HJ , Sabel M , Kamp MA . Recurrence pattern analysis of primary glioblastoma. World Neurosurg. 2017;103:733‐740. doi:10.1016/j.wneu.2017.04.053 28434963

[32] Jain KK . A critical overview of targeted therapies for glioblastoma. Front Oncol. 2018;8:419. doi:10.3389/fonc.2018.00419 30374421 PMC6196260

[33] Sanders S , Debinski W . Challenges to successful implementation of the immune checkpoint inhibitors for treatment of glioblastoma. Int J Mol Sci. 2020;21(8):2759. doi:10.3390/ijms21082759 PMC7215941 32316096

[34] Kim K , Fisher MJ , Xu SQ , El‐Deiry WS . Molecular determinants of response to TRAIL in killing of normal and cancer cells. Clin Cancer Res. 2000;6(2):335‐346. 10690508

[35] Bagci‐Onder T , Agarwal A , Flusberg D , Wanningen S , Sorger P , Shah K . Real‐time imaging of the dynamics of death receptors and therapeutics that overcome TRAIL resistance in tumors. Oncogene. 2013;32(23):2818‐2827. doi:10.1038/onc.2012.304 22824792 PMC3676868

[36] Kuijlen JMA , Bremer E , Mooij JJA , Den Dunnen WFA , Helfrich W . Review: on TRAIL for malignant glioma therapy? Neuropathol Appl Neurobiol. 2010;36(3):168‐182. doi:10.1111/J.1365-2990.2010.01069.X 20102513

[37] Stuckey DW , Shah K . TRAIL on trial: preclinical advances in cancer therapy. Trends Mol Med. 2013;19(11):685‐694. doi:10.1016/j.molmed.2013.08.007 24076237 PMC3880796

[38] Redjal N , Zhu Y , Shah K . Combination of systemic chemotherapy with local stem cell delivered S‐TRAIL in resected brain tumors. Stem Cells. 2015;33(1):101‐110. doi:10.1002/STEM.1834 25186100 PMC4270944

[39] Lim SM , Kim TH , Jiang HH , et al. Improved biological half‐life and anti‐tumor activity of TNF‐related apoptosis‐inducing ligand (TRAIL) using PEG‐exposed nanoparticles. Biomaterials. 2011;32(13):3538‐3546. doi:10.1016/j.biomaterials.2011.01.054 21306770

[40] Shi D , Xu X , Ye Y , et al. Photo‐cross‐linked scaffold with kartogenin‐encapsulated nanoparticles for cartilage regeneration. ACS Nano. 2016;10(1):1292‐1299. doi:10.1021/acsnano.5b06663 26757419

[41] Wolberg AS . Thrombin generation and fibrin clot structure. Blood Rev. 2007;21(3):131‐142. doi:10.1016/j.blre.2006.11.001 17208341

[42] Bozec L , Odlyha M . Thermal denaturation studies of collagen by microthermal analysis and atomic force microscopy. Biophys J. 2011;101(1):228‐236. doi:10.1016/j.bpj.2011.04.033 21723833 PMC3127184

[43] Wong ML , Wong JL , Vapniarsky N , Griffiths LG . In vivo xenogeneic scaffold fate is determined by residual antigenicity and extracellular matrix preservation. Biomaterials. 2016;92:1‐12. doi:10.1016/j.biomaterials.2016.03.024 27031928 PMC5289067

[44] Puckert C , Tomaskovic‐Crook E , Gambhir S , Wallace GG , Crook JM , Higgins MJ . Molecular interactions and forces of adhesion between single human neural stem cells and gelatin methacrylate hydrogels of varying stiffness. Acta Biomater. 2020;106:156‐169. doi:10.1016/j.actbio.2020.02.023 32084598

[45] Li L , Yang M , Wang C , et al. Effects of cytokines and chemokines on migration of mesenchymal stem cells following spinal cord injury. Neural Regen Res. 2012;7(14):1106‐1112. doi:10.3969/j.issn.1673-5374.2012.14.010 25722702 PMC4340025

[46] Xu F , Shi J , Yum B , Ni W , Wu X , Gu Z . Chemokines mediate mesenchymal stem cell migration toward gliomas in vitro. Oncol Rep. 2010;31(12):1265‐1270. doi:10.3892/or 20428810

[47] Vogel C , Marcotte EM . Insights into the regulation of protein abundance from proteomic and transcriptomic analyses. Nat Rev Genet. 2012;13(4):227‐232. doi:10.1038/nrg3185 22411467 PMC3654667

[48] Sheets KT , Ewend MG , Mohiti‐Asli M , et al. Developing implantable scaffolds to enhance neural stem cell therapy for post‐operative glioblastoma. Mol Ther. 2020;28(4):1056‐1067. doi:10.1016/j.ymthe.2020.02.008 32109370 PMC7132621

[49] Crommentuijn MHW , Maguire CA , Niers JM , et al. Intracranial AAV‐sTRAIL combined with lanatoside C prolongs survival in an orthotopic xenograft mouse model of invasive glioblastoma. Mol Oncol. 2016;10(4):625‐634. doi:10.1016/j.molonc.2015.11.011 26708508 PMC4826802

[50] Wang S‐S , Feng L , Hu BG , et al. miR‐133a promotes TRAIL resistance in glioblastoma via suppressing death receptor 5 and activating NF‐κB signaling. Mol Ther Nucleic Acids. 2017;8:482‐492. doi:10.1016/j.omtn.2017.07.015 28918048 PMC5560119

[51] Wakimoto H , Kesari S , Farrell CJ , et al. Human glioblastoma‐derived cancer stem cells: establishment of invasive glioma models and treatment with oncolytic herpes simplex virus vectors. Cancer Res. 2009;69(8):3472‐3481. doi:10.1158/0008-5472.CAN-08-3886 19351838 PMC2785462

