# Supplementary material for: Use of FLOSEAL® as a scaffold and its impact on induced neural stem cell phenotype, persistence, and efficacy
Source: Bioeng Transl Med. 2022 Jan 21;7(2):e10283. doi: 10.1002/btm2.10283 (PMC9115686; doi:10.1002/btm2.10283)
Supplement: Supplementary file 1 — Appendix S1: Supporting information [file BTM2-7-e10283-s001.rtf]

Category	Gene	Assay ID	
NSC	PAX6
SOX9
C-MYC
NESTIN	Hs01088114_m1
Hs00165814_m1
Hs00153408_m1
Hs04187831_g1	
Pluripotency	NANOG
OCT4
PODXL2	Hs02387400_g1
Hs04260367_gH
Hs00210532_m1	
Differentiation	GFAP
TUBB3
VMAC	Hs00909233_m1
Hs00801390_s1
Hs00418522_m1	
Migration	PIK3CD
CSPG4
CD44
GDNF
SOX2
FGFR2
P2RX7
SDC1
TNC
STC1
IL-6R
HIF-1??
VCAM-1
PLAUR
FLT1
CXCR4	Hs00192399_m1
Hs00361541_g1
Hs01075864_m1
Hs01931883_s1
Hs01053049_s1
Hs01552918_m1
Hs00175721_m1
Hs04966523_m1
Hs01115665_m1
Hs00174970_m1
Hs01075664_m1
Hs00153153_m1
Hs01003372_m1
Hs00958880_m1
Hs01052961_m1
Hs00607978_s1	
Proliferation	KI67
IL-1R	Hs04260396_g1
Hs00991010_m1	
Other	TRAIL
HSPA5	APH6DPA
Hs00607129_gH	
Supplemental Table 1. qRT-PCR Assay Identification NumbersTable 2.1. qRT-PCR Assay Identification Numbers.
